# Fiber Preparation from Micronized Oat By-Products: Antioxidant Properties and Interactions between Bioactive Compounds

**DOI:** 10.3390/molecules27092621

**Published:** 2022-04-19

**Authors:** Dariusz Dziki, Urszula Gawlik-Dziki, Wojciech Tarasiuk, Renata Różyło

**Affiliations:** 1 Poland Department of Thermal Technology and Food Process Engineering, University of Life Sciences in Lublin, Głęboka 31, 20-612 Lublin, Poland; dariusz.dziki@up.lublin.pl; 2 Fibrecare Sp. z o.o., Słowackiego 16, 40-094 Katowice, Poland; w.tarasiuk@pb.edu.pl; 3 Department of Biochemistry and Food Chemistry, University of Life Sciences in Lublin, Skromna 8, 20-704 Lublin, Poland; 4 Faculty of Mechanical Engineering Bialystok, Bialystok University of Technology, Wiejska 45A, 15-351 Białystok, Poland; 5 Department of Food Engineering and Machines, University of Life Sciences in Lublin, Głęboka St. 28, 20-950 Lublin, Poland; renata.rozylo@up.lublin.pl

**Keywords:** oat by-products, micronization, phenolic acids, antioxidant properties, fibre, isobolographic analysis

## Abstract

This study aimed to investigate the possibility of utilizing oat by-products for fiber preparation. Oat husk (OH) and oat bran (OB) were micronized and used to prepare a novel product rich in fiber and with enhanced antioxidant properties. The basic chemical composition and phenolic acid profile were determined in OH and OB. The antioxidant properties of OH and OB were also analyzed. The type and strength of interactions between the biologically active compounds from their mixtures were characterized by an isobolographic analysis. The analyses showed that the sum of phenolic acids was higher in OH than in OB. Ferulic acid was dominant in both OH and OB; however, its content in OH was over sixfold higher than that in OB. The results also suggested that both OH and OB can be used for preparing fiber with enhanced antioxidant properties. The optimal composition of the preparation, with 60–70% of OH and 30–40% of OB, allows for obtaining a product with 60–70% fiber and enhanced antioxidant activity due to bioactive substances and their synergistic effect. The resulting product can be a valuable additive to various food and dietary supplements.

## 1. Introduction

Cereals have been an important component of a daily diet for centuries. In particular, the high consumption of fiber-rich cereal products has been shown to reduce the risk of several diseases [1,2]. The protective effect of such products is mainly attributed to dietary fiber [3] and polyphenols [4].

Oat (*Avena sativa* L.) is a valuable cereal crop in the developing world. Until recently, oat was primarily used as animal feed and, to some extent, as human food. Of late, oat has been gaining interest as a health food for humans, and its use as animal feed is steadily declining. Due to its nutritional benefits, as revealed by researchers around the world, oats are currently used in the food industry as an ingredient in various food products, including infant foods, bread, beverages, breakfast cereals, biscuits, and muesli, and also sold in the form of oat milk and oat flour [5].

Another reason for the growing popularity of oats is that their cultivation requires lesser nutrients than other cereals [6]. Oat is predominantly grown in American and European countries, mainly Russia and Canada [7]. Oat consumption by humans has been increasing because of the awareness of the health benefits of dietary fiber such as β-glucan and bioactive phytochemicals. These compounds are known to reduce the risk of type 2 diabetes and cardiovascular diseases and decrease the level of cholesterol and glucose in the blood. β-Glucan can also attenuate glycemic response, increase satiety after a meal, and benefit gut microflora [8]. It is mainly found in the oat bran (OB) fraction [9]. In addition to β-glucan, the OB fraction contains various phenolic compounds, including ester-linked glycerol conjugates, ester-linked alkyl conjugates, ether- and ester-linked glycerides, anthranilic acid, and avenanthramide, with a high antioxidant capacity [10]. OB, which is a by-product obtained from the milling of oat flour, is relatively inexpensive and is believed to provide health benefits when added to food [11,12].

Kim and Dale [13] reported that the processing of crops (oats, wheat, rice, corn, or sorghum) results in approximately 1.5 billion metric tons of waste biomass worldwide. Although the waste occurs primarily in the form of straw, the operation of postharvest lines, which removes the remnants of native plants and husks from processed crops, generates a large amount of waste biomass that is unsuitable for transportation and combustion [14]. Oat husk (OH) is a by-product produced during oat processing for food purposes. It makes up about 25–33% of the weight of oat. Around 2.75–3.3 million tons of OH are generated each year by oat processing [15]. As a low-value lignocellulosic residue, OH can have environmental consequences. However, their polymers can be converted into several value-added products, but this requires efficient pretreatment methods for their fine separation for further valorization [16]. As a raw material rich in fiber with low energy and low protein, OH is primarily used as animal feedstock and biofuel [17]. Nevertheless, due to a very low bulk density of about 144 kg/m^3^, the handling of OH is also challenging [18,19]. On the other hand, OH is an excellent source of insoluble fiber, with a documented health effect on humans [20]. Its fiber is resistant to fermentation in the human colon, has no impact on serum lipids, and provides no energy to the body. The inclusion of insoluble fiber in the human diet can help maintain healthy colon function and reduce constipation [21].

The food industry is in constant search of novel sources of insoluble fiber. Traditionally, OH has been discarded during oat processing. Still, the need for concentrated, insoluble fiber sources for human consumption has paved the way for the production of oat hull fiber. Although the effects of oat hull fiber have been analyzed in animals such as rats, pigs, chickens, and cattle [22], studies focusing on the possibility of using OH as a food additive are very limited. Piwińska et al. [23] studied the effect of adding a mixture containing OH and soluble oat fraction to wheat pasta. Oliveira et al. [24] proved that OH could be a valuable cellulose fiber source for hydrogel production.

Due to the high fiber content, the traditional size reduction method is insufficient for grinding OH. Ultrafine grinding or micronization is a new technique used for making a super fine powder with a particle size of 1–100 μm and good surface properties [12,25]. This very fine powder is characterized by higher solubility, dispersibility, and water absorption, which improves the quality of the target food products. Moreover, micronization considerably enhances the efficiency of extraction of phytochemicals [20], and is widely employed to extract natural polysaccharides from different bioresources [26].

This study aimed to investigate the possibility of utilizing micronized OB and OH to prepare a new fiber-rich product with enhanced antioxidant properties. In addition, the study analyzed the interactions between the biologically active compounds from OH and OB.

## 2. Results and Discussion

### 2.1. Basic Composition of Raw Materials

Table 1 presents the results of the basic chemical composition of OH and OB. Compared to OB, OH had a higher content of ash (3.41 and 2.74 g/100 g in OH and OB, respectively). It was also fat-free, whereas OB had 5.52% of fat. OB was characterized by a several-fold higher protein content (16.30%) than OH (1.31%). This is in line with a previous study [27] that showed that OH protein content does not exceed 4%. Furthermore, OB contained 6.05 g/100 g dry weight (DW) of β-glucan, whereas in OH, the amount of this compound was only 0.25 g/100 g DW. Higher total fiber content was found in OH (91.11 g/100 g DW) than in OB (23.60 g/100 g DW). A similar composition of OH and OB was reported by Dziki et al. [20] and Xue et al. [12], respectively.

OH is especially rich in insoluble fiber such as cellulose, hemicelluloses, and lignin [24], whereas both soluble and insoluble fiber occurs in OB in a ratio of 1:5 [28]. It is worth emphasizing that OB has a higher soluble dietary fiber content (especially β-glucan) than wheat or rice bran [28]. Soluble dietary fiber has many health effects, including preventing cardiovascular diseases, diabetes, and obesity [29]. On the other hand, insoluble dietary fiber supports normal intestinal peristalsis [30]. Moreover, consumption of insoluble fiber-rich products can help to reduce appetite and food intake [31].

### 2.2. Identification and Quantification of Phenolic Compounds

Phenolic acids are mainly located in the outer part of the cereal grain. The content of these compounds is almost 15–18-fold higher in the bran compared to the endosperm [32,33]. Due to the presence of hydroxyl groups and phenolic rings, phenolic acids can exhibit antioxidant activity (AA), which is one of their most important properties [33]. As shown in Table 2, both OH and OB contained a significant amount of phenolic acids. The sum of phenolic acids was higher in OH than in OB (456.58 and 151.98 µg/mg DW, respectively). In both OH and OB, the dominant phenolic compound was ferulic acid. However, its content in OH was more than sixfold higher than in OB. In OH, ferulic acid accounted for more than 95% of all phenolic acids, whereas in OB, it constituted about 47%. An earlier study [34] also confirmed that ferulic acid was the major phenolic acid in OH. Sevgi et al. [35] showed that ferulic acid exhibited the highest AA compared to other phenolic acids such as p-hydroxybenzoic, caffeic, gallic, protocatechuic, vanillic, and rosmarinic acid. OB also contained a high amount of p-coumaric acid (61.53 µg/mg DW), whereas, in OH, the content of this acid was above the limit of detection and below the limit of quantification. Caffeic acid was present in similar amounts in both OH and OB (6.08 and 5.72 µg/mg DW, respectively). OH was also richer in protocatechuic, p-hydroxybenzoic, and vanillic acids than OB, while OB contained a higher amount of synaptic acid (6.55 µg/mg DW). Salicylic acid was found in a small proportion only in OB (0.09 µg/mg DW).

It was shown that 1 g of OH contained 269.9 µg of p-coumaric acid, 309 µg of ferulic acid, 11.2 µg of vanillic acid, 1.4 µg of sinapic acid, 6.8 µg of syringic acid, and 10.9 µg of *p*-hydroxybenzoic acid [34]. These values differ from those estimated in our study, which may be due to genetic factors and the method of extraction. A study [36] showed that oat grain is rich in the following phenolic acids: *p*-hydroxybenzoic, dihydroxybenzoic, caffeic, *p*-coumaric, ferulic, vanillic, sinapic, gallic, and syringic acid. OH contains about fourfold higher ferulic acid content than oat grain [34]. Dziki et al. [20] determined a similar amount of phenolic acids in micronized OH. Hitayezu et al. [4] found that OB contained five main phenolic acids: vanillic, caffeic, *p*-coumaric, ferulic, and cinnamic acid. The authors observed ferulic acid constituted about 64% of all phenolic acids in the fine bran fraction. They also noted that lower granulation of bran contributed to the improved extraction of phenolic acids.

### 2.3. Total Phenolic Content (TPC) and AA of OH and OB

**Figure 1:**
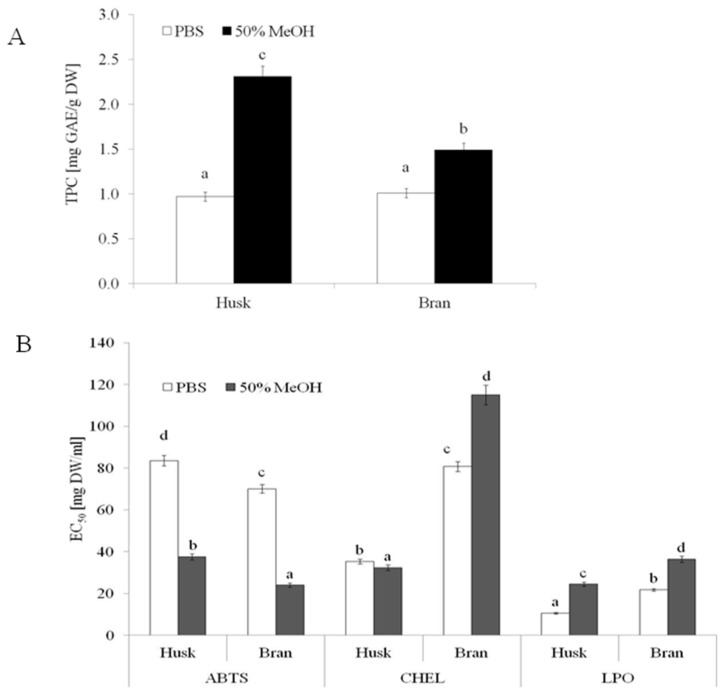
TPC (**A**) and AA (**B**) of micronized OB and OH. PBS—buffer extract; 50% MeOH—hydroalcoholic extract; ABTS—antiradical activity; CHEL—chelating power; LPO—ability to protect lipids against oxidation. Means followed by different lowercase letters (a–d) are significantly different at *p* < 0.05.

Several methods can evaluate the phenolic content and AA of plant foods. The analytical technique involves using nonspecific methods to determine the overall content of phenolic compounds, which is usually expressed as an index such as gallic acid, chlorogenic acid, or catechin equivalent [37]. As presented in Figure 1B, both OH and OB contained comparable amounts of buffer-extractable phenolics (0.97 and 1.01 mg gallic acid equivalent (GAE)/g DW for OH and OB, respectively), whereas a significantly higher TPC was found in the hydroalcoholic extract of OH (2.31 mg GAE/g DW) compared to that of OB (1.47 mg GAE/g DW). A similar level of phenolics (2.6 mg/g DW) was found in OH by other authors after its extraction with 75% aqueous methanol [34]. By contrast, Călinoiu and Vodnar [33] showed a lower TPC in OB (0.25 mg GAE/g DW) extracted with 80% methanol using an ultrasonic bath. The content of extracted phenolic compounds depends on the extraction method used and the particle size of raw materials. A higher degree of fineness of OB and OH is associated with higher TPC [4,20].

Most studies investigating the anti-free-radical scavenging activity of oats have used the DPPH (2,2-diphenyl-1-picrylhydrazyl) assay [38,39,40]. However, a study [41] indicated that the ABTS (2,2′-azino-bis(3-ethylbenzothiazoline-6-sulfonic acid)) assay may also be used to determine the activity of both hydrophilic and hydrophobic antioxidants. This study also indicated that ABTS is not influenced by the ionic strength and reacts with most of the antiradical compounds. The results obtained for different food samples suggested that the ABTS assay better estimates the antioxidant content than the DPPH assay [37]. Higher AA was found in hydroalcoholic extracts of both OB and OH (Figure 1A). The extracts of both raw materials exhibited significant ABTS^•+^-quenching ability, and the hydroalcoholic extracts of both extracts showed more than twofold higher radical quenching activity. Regardless of the type of extract, the samples obtained from OB were characterized by higher ABTS^•+^ quenching ability. The highest AA was found in hydroalcoholic extracts from OB (EC_50_ = 24.07 mg DW/mL). Phenolic compounds mainly determine the AA of OH and OB extracts; however, the amount of phenolic acids, rather than the composition of extracts (the type of phenolic compounds and their proportion), seems to play a key role in the AA of the extracts.

Kruma et al. [37] showed that hulled oats exhibited significantly higher ABTS scavenging activity than hull-less oats. Other authors [42] demonstrated that the insoluble phenolic fraction of oat showed significantly higher AA (ABTS) compared to the free phenolic fraction. AA was determined by both the method of sample preparation and extraction procedure. Liu et al. [26] proved that the antiradical activity of the polysaccharide extracts of OB obtained via superfine grinding was significantly higher than that of extracts obtained from coarse particles of OB. Notably, the ABTS scavenging activity of the extracts increased from 38.87% to 62.29%. Considering the chelating power (CHEL), a significantly higher AA (lower EC_50_) was observed for the OH extracts than for the OB extracts.

The OH extract containing the hydroalcoholic extractable compounds was characterized by lower EC_50_ values compared to buffer extracts (EC_50_ = 32.4 and 35.37 mg DW/mL, respectively). An opposite trend was observed in the case of OB extracts, in which a significantly higher activity was observed compared to buffer-extractable compounds (EC_50_ = 80.73 and 115.03 mg DW/mL for buffer and hydroalcoholic extract, respectively). Metals such as Fe^2+^ (in the free form) can participate in the Fenton reaction, generating hydroxyl radicals. HO∙ radicals are characterized by the strongest reactivity and oxidation power than other reactive oxygen species (ROS). Thus, substances that can chelate free Fe^2+^ ions are critical in reducing HO∙radicals and associated damage [43].

Another process that has a deleterious effect on foods and is most damaging to living organisms is lipid peroxidation [1]. Interesting results were observed by analyzing the ability of products to inhibit lipid oxidation. In this study, the OH extracts, regardless of the type, showed a higher ability to inhibit lipid peroxidation compared to OB extracts. This suggests that both raw materials contain potentially bioaccessible compounds that can effectively inhibit lipid oxidation (Figure 1B). In fact, molecules with higher peroxyl radical (ROO) scavenging activity do not often exhibit higher metal chelating properties. This is because the chelating activity is determined by the binding characteristics of the active molecule. In contrast, the ROO∙ activity depends on the ability of a molecule to transfer electrons or protons [4]. Phenolic acids, including ferulic, caffeic, p-coumaric, and cinnamic acids, have been reported to differently inhibit the oxidation of linoleic acid, with ferulic acid being the most active. Phenolic acids identified in OB extracts certainly contributed to their activity [1].

### 2.4. Interaction Assay

**Figure 2:**
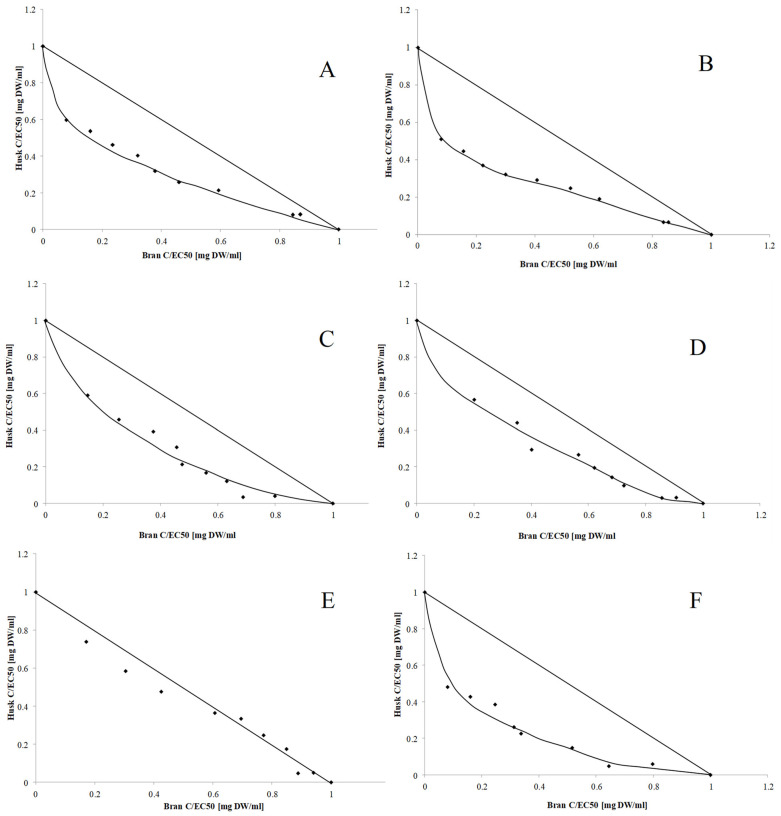
Dose-normalized isobolograms for the antioxidant activity of OH and OB components: antiradical activity of buffer extract (**A**) and 50% hydroalcoholic extract (**B**); CHEL of buffer extract (**C**) and 50% hydroalcoholic extract (**D**); and lipid peroxidation-inhibiting ability of buffer extract (**E**) and 50% hydroalcoholic extract (**F**).

The interaction between bioactive components influences the final activity of their mixture [44]. Thus, this study analyzed the strength of interactions occurring between the biologically active compounds from OH and OB. In the first step of the analysis, the type and strength of interactions were determined using normalized isobolograms. As shown in Figure 2, synergism was observed between compounds that indicated their antiradical activity and CHEL regardless of the type of extract, while buffer-extractable compounds additionally exhibited the ability to inhibit lipid peroxidation. Synergism was found in 50% of methanol-extractable compounds that could prevent lipid oxidation. Considering the beneficial interactions between antioxidant compounds, it seemed justified to prepare a mixture of OH and OB and evaluate its antioxidant properties.

To determine the optimal composition of the OH-OB mixture, the combination index (CI) of each of the tested mixtures was determined (Table 3). The best antiradical activity was observed in the samples with the highest proportion of OH (60–90%), among which the higher activity was noted for hydroalcoholic extracts. These observations and the average CI values clearly indicated the synergism between antiradical compounds present in both OH and OB. A higher metal-chelating ability was observed in the samples containing at least 50% OB. The average CI values determined in both extracts indicated synergism between active compounds. The solvent used for extraction did not seem to affect the activity of the tested samples.

The analysis of the ability to inhibit lipid peroxidation yielded interesting results. For buffer-extractable phytochemicals, the highest activity was found in the samples containing a higher proportion of bran, while this relationship was not observed for hydroalcoholic extracts. Moreover, the average CI value indicated the synergism of buffer-extractable phytochemicals and compounds extracted by 50% MeOH.

Based on the CI value, compounds with the highest activity were selected. As presented in Table 4, the CI index for the selected composition differed from the average value. Taking into account the antiradical activity, the highest effect was observed in the mixtures containing 90% OH (extracted using phosphate-buffered saline (PBS) buffer) and 70% and 90% OH (extracted using 50% methanol). In terms of the ability to chelate transition metal ions, in the case of PBS buffer extract, the highest activity was noted for the mixture containing equal proportions of both components, while the mixture with 70% OH exhibited the highest activity among the 50% methanol extracts. Analysis of the influence of the mixture composition on the ability to inhibit lipid peroxidation revealed that the mixture containing 60% husk had the optimal composition.

## 3. Materials and Methods

### 3.1. Chemicals

All the chemicals used were of analytical grade. DPPH, ABTS, Folin–Ciocalteu reagent (2 N), methanol, linoleic acid, ammonium thiocyanate, gallic acid, and ferrozine were purchased from Sigma-Aldrich (Poznan, Poland). Acetonitrile (high-performance liquid chromatography-grade) was purchased from Merck (Darmstadt, Germany). Kaempferol was purchased from Fluka AG (Buchs, Switzerland). Formic acid (liquid chromatography-mass spectrometry-grade) was obtained from Merck (Darmstadt, Germany). A purification system (Milli-Q-Simplicity-185, Millipore Corp., Burlington, MA, USA) was used for obtaining ultrapure water.

### 3.2. Plant Materials

The plant raw materials used in the study were OB and OH. OB were purchased from ZPZM Kruszwica Sp. z o.o. (Kruszwica, Poland), and OH was purchased from AG Feeding Sp. z o.o. (Gdynia, Poland). Before their use, both raw materials were sterilized and micronized, as described previously [20,45].

### 3.3. Determination of Basic Chemical Composition

The basic composition of OB and OH was determined using the standard methods as follows [AOAC, 2010] [46]: moisture content—Method 925.10, protein content—Method 992.33 (Nx6.25), ash content—Method 942.05, fat content—Method 30–10, and β-glucan content—Method 995.16. 

### 3.4. Phenolic Acid Analysis

For phenolic acid analysis, the UPLC-MS/MS (ultra-performance liquid chromatography-mass spectrometry) method was used. Pulverized samples of OB and OH were analysed and calculated according to the method described by Dziki et al. [20].

### 3.5. TPC and AA

#### 3.5.1. Extract Preparation

To study the antioxidant properties of OH, OB, and their mixtures (OH with OB: 9:1, 8:2, 7:3, 6:4, 5:5, 4:6, 3:7, 2:8 and 1:9), their buffer extract (phosphate-buffered saline) and 50% methanol extract were prepared [47,48,49].

#### 3.5.2. TPC Estimation

The TPC of the extracts obtained from OH and OB and their mixtures was determined as described by Singleton et al. [50] with slight modifications [51]. The values were expressed as GAE/g DW.

#### 3.5.3. Antiradical Activity (ABTS)

The ABTS^•+^-quenching ability of OH and OB and their mixtures was determined as described previously [52] using the following equation:

SC = [(A_C_ − A_A_)/A_C_)] × 100% (1)

where SC is scavenging ability, A_C_ is the absorbance of the control, and A_A_ is the absorbance of the sample.

#### 3.5.4. Metal-Chelating Activity (CHEL)

The metal-chelating activity (CHEL) of OH and OB and their mixtures was determined as described previously [53] using the following formula:

IN = [1 − (A_S_/A_C_)] × 100% (2)

where IN is inhibiting ability, As is the absorbance of the sample, and Ac is the absorbance of the control.

#### 3.5.5. Inhibition of Linoleic Acid Peroxidation

The inhibition of linoleic acid peroxidation was determined as described previously [54], but using an aqueous solution of 10 mmol/L FeCl_2_ instead of hemoglobin.

#### 3.5.6. AA Determination

For all the assays used to determine the AA of OH and OB and their mixtures, the half-maximal inhibitory concentration or EC50 values were calculated by interpolating the dose-response curves. The EC_50_ values were calculated in fitted models as the concentration at which the tested compound exhibited 50% of the maximum inhibition based on a dose-dependent mode of action.

### 3.6. Interaction Analysis

The type and strength of interactions between biologically active compounds from OH and OB mixtures were determined by isobolographic analysis based on CI values proposed by Chou [55]. The CI value at which the drug combination exhibited x% inhibition was calculated as follows [55]:

(3) $$\mathrm{CI}=\frac{{\left(D\right)}_{1}}{{\left(D_{x}\right)}_{1}}+\frac{{\left(D\right)}_{2}}{{\left(D_{x}\right)}_{2}}$$

where CI is the sum of the dose of the components that exert x% inhibition when combined and Dx is the dose (D) as a single substance that inhibits a system at x%. A CI value of <1, >1, and 1 indicates that the type of interaction is synergistic, antagonistic, and additive, respectively. OH and OB were mixed in ratios for the interaction analysis as described in the “Results and Discussion” section.

### 3.7. Statistical Analyses

All tests were performed in triplicate unless stated otherwise. The results were presented as mean values and standard deviations. The data were also subjected to a one-way analysis of variance, and Tukey’s test determined the differences between means. The significance level (α) was established at 0.05.

## 4. Conclusions

The obtained results justify the use of OH as a hitherto underappreciated ingredient in the production of fiber preparations with enhanced antioxidant properties. The optimal composition of the micronized oat preparation containing 60–70% OH and 30–40% OB can allow the obtainment of a product rich in fiber (about 60%) with exceptional health properties and high AA due to the presence of bioactive substances from both husk and bran, as well as their synergistic effect. Such a product can be a valuable additive for various food products such as bread, pastry, and pasta. Appropriate fragmentation with micronization enables the use of the preparation in the dairy industry and the production of beverages. Such highly fragmented preparation can also be applied in the pharmaceutical industry as an additive to dietary supplements.

## Figures and Tables

**Table 1:** Comparison of The Basic Composition of Oat Husk and Bran (g/100 g DW).

Parameter	Husk	Bran
Moisture content	3.5 ± 0.12	4.2 ± 0.08
Ash content	3.41 ± 0.10	2.74 ± 0.16
Protein content	1.31 ± 0.08	16.30 ± 0.29
Fat content	nd *	5.52 ± 0.28
β-glucans content	0.25 ± 0.04	6.05 ± 0.25
Total fiber content	91.11 ± 1.35	23.60 ± 1.7
Total carbohydrates	91.90 ± 1.63	69.4 ± 1.10

* Not detected.

**Table 2:** Comparison of The Basic Composition of Oat Husk and Bran (g/100 g DW).

Phenolic Acid	Husk	Bran
Caffeic	6.08 ± 0.20	5.72 ± 0.04
Ferulic	435.71 ± 20	70.74 ± 0.86
*p*-coumaric	>LOQ	61.53 ± 0.89
*p*-hydroxybenzoic	4.98 ± 0.12	3.14 ± 0.06
Protocatechuic	0.71 ± 0.01	0.22 ± 0.00
Salicylic	<LOD	0.09 ± 0.01
Sinapic	1.82 ± 0.12	6.55 ± 0.15
Vanillic	4.27 ± 0.15	1.59 ± 0.06
Syringic	3.01 ± 0.35	2.40 ± 0.17
Sum	456.58 ± 19.90	151.98 ± 1.58

>LOQ—above the limit of detection and below the limit of quantification, <LOD—below the limit of detection.

**Table 3:** Antioxidant Activity of OB and OH Mixtures and Combination Index.

Mixture OB:OH	Antiradical Activity				Metal-Chelating Activity				Inhibition of Lipid Peroxidation			
	PBS		50% MeOH		PBS		50% MeOH		PBS		50% MeOH	
	EC_50_ *	CI	EC_50_	CI	EC_50_	CI	EC_50_	CI	EC_50_	CI	EC_50_	CI
9:1	67.8 ± 0.3 ^d**^	0.95	25.2 ± 1.8 ^c^	0.90	32.4 ± 0.8 ^b^	0.84	36.4 ± 1.0 ^a^	0.94	10.7 ± 0.2 ^a^	0.94	17.5 ± 0.5 ^a^	0.69
8:2	65.8 ± 2.7 ^d^	0.92	25.0 ± 0.8 ^c^	0.92	27.8 ± 0.6 ^a^	0.72	34.7 ± 1.1 ^a^	0.88	11.0 ± 0.1 ^a^	0.99	21.7 ± 1.6 ^c^	0.85
7:3	59.5 ± 0.8 ^c^	0.80	23.8 ± 0.9 ^c^	0.80	33.4 ± 1.2 ^b^	0.75	37.4 ± 0.9 ^a^	0.82	12.8 ± 0.2 ^b^	1.02	18.1 ± 0.7 ^ab^	0.66
6:4	53.8± 1.3 ^a^	0.71	21.4 ± 0.7 ^ab^	0.76	33.8 ± 0.4 ^b^	0.73	41.3 ± 0.8 ^b^	0.82	13.5 ± 0.8 ^b^	1.02	21.6 ± 0.8 ^c^	0.76
5:5	53.2 ± 0.6 ^a^	0.70	20.0 ± 0.6 ^ab^	0.69	34.5 ± 0.9 ^b^	0.68	45.7 ± 2.1 ^c^	0.81	14.6 ± 0.1 ^cb^	1.03	17.5 ± 0.4 ^a^	0.59
4:6	56.1 ± 1.3 ^b^	0.72	20.1 ± 0.7 ^ab^	0.62	41.6 ± 1.0 ^c^	0.76	52.0 ± 1.6 ^d^	0.83	14.5 ± 0.3 ^cb^	0.88	19.1 ± 0.8 ^b^	0.57
3:7	55.2 ± 0.7 ^ab^	0.69	19.9 ± 0.3 ^a^	0.59	45.5 ± 0.2 ^d^	0.76	48.7 ± 6.2 ^dc^	0.69	14.9 ± 0.1 ^c^	0.90	18.1 ± 0.7 ^ab^	0.66
2:8	55.9 ± 0.6 ^ab^	0.69	20.8 ± 0.7 ^ab^	0.60	46.8 ± 0.4 ^d^	0.71	63.5 ± 1.0 ^e^	0.79	16.8 ± 0.8 ^d^	0.93	19.5 ± 0.7 ^b^	0.59
1:9	55.5 ± 0.3 ^b^	0.68	21.4 ± 0.8 ^ab^	0.59	52.6 ± 1.2 ^e^	0.74	72.2 ± 1.8 ^f^	0.76	18.0 ± 0.2 ^d^	0.91	21.5 ± 0.3 ^c^	0.61

* EC_50_—half maximal inhibitory concentration, CI—combination index, ^**^ Means in rows followed by different lowercase letters (a–e) are significantly different at *p* < 0.05.

**Table 4:** The Best Connections OB:OH for The Tested Antioxidant Activities, Combination Index (CI), and The Type of Interaction.

Antioxidant Assay	Kind of Extract	Average CI	Type of Interaction	Composition OB:OH	The Best CI	Type of Interaction
ABTS *	PBS 50% MeOH	0.78 0.72	moderate synergism moderate synergism	1:9	0.68	synergism
				3:7; 1:9	0.59	synergism
CHEL	PBS 50% MeOH	0.74 0.81	moderate synergism moderate synergism	1:1	0.68	synergism
				3:7	0.69	synergism
LPO	PBS 50% MeOH	0.96 0.66	Addition synergism	4:6	0.88	slight synergism
				4:6	0.57	synergism

* ABTS—ability to quench ABTS^•+^ radicals; CHEL—metal-chelating activity, LPO—inhibition of linoleic acid peroxidation.

## Data Availability

The data presented in this study are available upon request from the corresponding author.
